# Barriers and Enablers of Postnatal Care by Accredited Social Health Activist (ASHA) Workers: A Community-Based Qualitative Study From Tribal Gujarat

**DOI:** 10.7759/cureus.56667

**Published:** 2024-03-21

**Authors:** Kinjal Gadhavi, Niraj Pandit, Neelabh Pankaj

**Affiliations:** 1 Department of Community Medicine, Smt. B. K. Shah Medical Institute & Research Centre, Sumandeep Vidyapeeth (Deemed to be University), Vadodara, IND

**Keywords:** home visit, newborn, postnatal mother, asha, postnatal care

## Abstract

Background

The care provided to the mother and child from delivery to six weeks after is defined as postnatal care. The postnatal period is both a happy and critical phase for the mother and the newborn. However, the provision of high-quality care services is often ignored during this time. The objective of this study was to assess postnatal care services quality by Accredited Social Health Activist (ASHA) workers and associated factors such as newborn care in rural tribal areas of Gujarat, India.

Methodology

An ethnographic approach was adopted. Four primary health centers (PHCs) were selected purposively from Sankheda Block, Chhotaudepur, a tribal district in the eastern part of Gujarat. Information on obstacles and facilitators of postnatal care services was collected using in-depth interviews (IDIs) with a purposive sample of 22 ASHAs working in selected PHCs. Qualitative data were analyzed using thematic analysis.

Results

The median age of the ASHA workers was 39 years and ranged from 30 to 51 years (N = 22). Most ASHAs encountered logistical challenges when offering postnatal care services (e.g., they struggled to care for the mother and her babies because they were missing essential equipment, such as a thermometer and a salter-type baby weighing machine, or they had broken equipment). The two main issues facing ASHAs were incentives and timely payments. There were concerns about their safety and physical security during fieldwork. The majority of ASHA workers had good experiences during postnatal home visits, and they received support from other healthcare workers. There were many misconceptions and false assumptions in the community regarding breastfeeding, prelacteal feeding, family planning, and contraception methods. ASHAs wanted to become long-term government employees and believed they were entitled to sufficient training, assistance, recognition, and remuneration for the duties they performed.

Conclusions

Postnatal mothers receive considerably less attention than antenatal mothers because it mostly depends on ASHA workers and field staff. ASHA workers are doing their best regarding postnatal care. This study revealed some issues ASHAs face, including logistic issues, transportation issues, regular and timely payment issues, and local-level acceptance issues.

## Introduction

The care provided to the mother and the child from delivery to six weeks after is defined as postnatal care [1]. The postnatal period is a happy and critical phase for the mother and the newborn. However, the provision of high-quality care services is often ignored during this period. Most deaths occur during this period [2].

According to Approach for Mother, Children and Adolescent Health (2016-2030), Strategies Toward Ending Preventable Maternal Mortality, Every Newborn Child Action Plan, and other initiatives, the postpartum period continues to carry the heavy burden of mother and newborn mortality and morbidity [3]. Maternal mortality is considered to be a key indicator of health, and the direct causes of maternal deaths are well-known and often preventable or treatable [4].

Postnatal care involves providing specialized services to women and their newborn children. Lack of care during this period may result in death or disability, as well as missed opportunities to promote healthy behaviors, affecting women and newborns. After giving birth, a mother experiences many physical and mental changes as she learns how to take care of her newborn. Postnatal care can include maternal nutrition, breastfeeding support, immunization, and interactive sessions on the value of birth spacing and the prevention, elimination, early identification, and treatment of health issues. Prompt and suitable postpartum care ensures significant improvements to both the mother and child’s health [5].

Low-middle-income countries like India face major challenges related to maternal and child healthcare because of inadequate services [6]. The National Health Mission aims to provide effective healthcare to rural residents, mainly poor women and children. To achieve this, female Accredited Social Health Activists (ASHAs) have been appointed to every village. ASHAs are directly involved in antenatal and postnatal care. They are grassroots workers and many of them work over eight hours a day, leaving them feeling fatigued and exhausted [7]. It may eventually become difficult for them to provide quality care. Several other countries have reported that assigning multiple tasks to healthcare workers can lead to role confusion, an inability to fulfill multiple roles, and difficulty managing priorities [8].

This study aims to assess the quality of postnatal care services delivered by ASHA workers and associated factors such as newborn care in rural tribal areas of Gujarat, India.

## Materials and methods

Study design and setting

This study was conducted in Chhotaudepur, a tribal district located in the eastern part of Gujarat. An ethnography approach was adopted to collect data on postnatal care service barriers and enablers among ASHA workers in four primary health centers (PHCs), i.e., Bahadarpur, Gundicha, Bhatpur, and Vasana, in Sankheda, a Block of Chhotaudepur. The total population of Sankheda Block is 203,584 [9].

**Figure 1:**
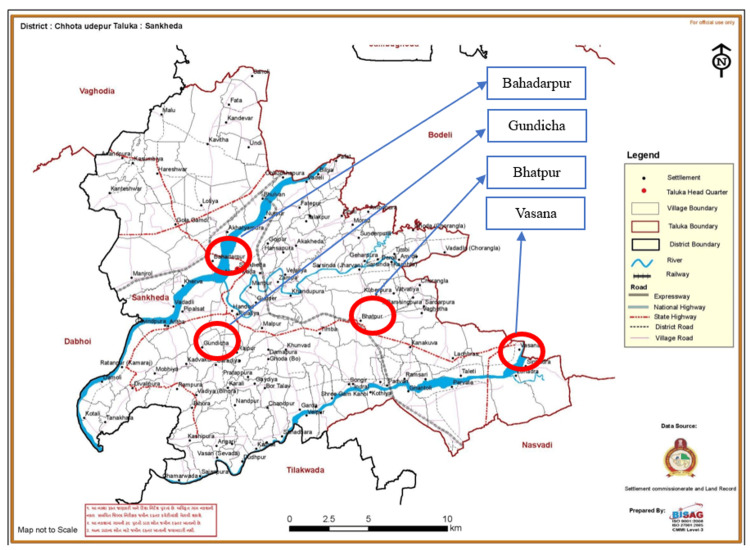
The four study sites in Sankheda Block, Chhotaudepur district, Gujarat. [10].

Figure 1 shows a map of the four PHCs of the Sankheda Block in Chhotaudepur district. Sankheda Block was selected randomly among all the blocks of the Chhotaudepur district. The areas highlighted with a red encircle on the map, namely, Bahadarpur, Gundicha, Bhatpur, and Vasana PHC, were selected as the study sites. Data were collected from all four PHCs.

Sample and sampling technique

A total of 22 ASHA workers were selected using the purposive sampling method. A community-based exploratory qualitative study was conducted. ASHAs were purposively selected from four PHCs, and data were collected from February to July 2022. The present study is part of a larger study titled “Evaluation of postnatal care of mothers including newborns in rural areas of Chhotaudepur district, Gujarat: A community-based mixed-methods study.” Interviews were conducted at the homes of the ASHA workers.

Data collection procedure

The qualitative interview method with a semi-structured interview guide was used to conduct in-depth interviews (IDIs) of ASHA workers. A total of 22 ASHAs participated. Written informed consent and consent for audio-recording the interviews were obtained. The interviews were conducted one-on-one, and care was taken to ensure that no third party was present during the interviews. Each interview lasted between 20 and 40 minutes. The number of interviews was determined by applying theoretical saturation principles.

Research instruments

A semi-structured interview guide was used to conduct IDIs with participants to better understand their experiences with PHC services. To ensure validity, the study tools were initially developed in English and validated with a group of public health experts. The interviews were conducted in Gujarati, the local language. The investigator audio-recorded the interviews, translated them into English, and then prepared a verbatim transcript.

Data analysis

Thematic analysis was conducted on data collected through the IDIs using NVivo 14.0 software. The transcripts were matched with the audio recordings to ensure accuracy. During the transcription process, the transcripts were read, interpreted, and coded. In reviewing the transcripts, investigators classified the coded data into major discussion points, allowing the main themes to emerge from each topic. The thematic consensus was double-checked for any inconsistencies before finalizing the themes.

Ethical approval

Ethical clearance for the study was obtained from the Institutional Ethical Committee, Sumandeep Vidyapeeth, Piparia, Waghodiya, Gujarat. Written and informed consent was obtained from each participant.

## Results

In total 22 interviews were conducted and themes were identified based on the response of the participants. A total of six main themes and 12 sub-themes (categories) were identified from the analysis of the interview transcripts.

As shown in Table 1, the average age of ASHA workers was 39.90 years. The majority of them were educated up to secondary school. Overall, 45.45% of ASHAs had work experience of 11-15 years.

**Table 1:** Demographic profile of ASHA healthcare workers (N = 22) ASHA = Accredited Social Health Activist

Variable	Category	Frequency	Percentage
Age (in years)	30–39	11	50%
	40–49	8	36.36%
	≥50	3	13.63%
Education	8th pass	4	18.18%
	9th pass	7	31.81%
	10th pass	7	31.81%
	12th pass	3	13.63%
	Graduate	1	4.54%
Work experiences (in years)	<5	3	13.63%
	5–10	9	40.90%
	11–15	10	45.45%

Theme 1: issue or problem in providing PHC care

Category 1.1: Logistic Issues

The majority of ASHA workers claimed that logistics were a problem. Because of a lack of necessary items or damaged equipment, such as baby weighing machines, thermometers, medicine, and contraception, they found it difficult to provide care for mothers and their newborns. The support received from village health and sanitation committees (VHSCs) was not satisfactory. According to the ASHAs, the VHSCs should help with the maintenance and purchase of required equipment. Additionally, PHCs and VHSCs should take responsibility for managing logistics themselves.

Category 1.2: High Workload and Poor Incentives

All ASHAs mentioned high workloads, poor incentives, and a lack of institutional recognition. These are the key determinants in providing optimal maternity care. Timely payments and insufficient incentives were the two major problems ASHAs raised. ASHA workers expected at least some fixed remuneration from the government in addition to incentives to keep up with inflation. They believed their workload was greater than that of female health workers (FHWs):

“We can’t afford this inflation with this poor incentive.” (ASHA 2)

“We are doing more than FHW.” (ASHA 3)

Category 1.3: Transportation-Related Issue

Typically, an ASHA should be responsible for only one village, but in our study group, an ASHA was responsible for multiple villages. The need to travel from village to village created a transportation problem. Security and safety were of concern to ASHAs. There was no support system in place to minimize transportation challenges. Further, village transportation is usually challenging. It was observed that frequent travel was a challenge for ASHAs:

“There won’t be a problem if the mother’s house is nearby, but there is a problem if I want to go to the visit mother’s house that is located far distant and don’t have a transportation or vehicle.” (ASHA 5)

Category 1.4: Social Stigma and Humiliation

ASHA workers faced discrimination not just in the public domain but also at home. They received several insulting comments from residents or members of the household during a postnatal home visit. They also frequently faced criticism for not performing their duties correctly. ASHAs also had to put up with harassment when on field visits:

“The mother who had failed the operation for family planning was pregnant. In the evening, when I visited her house, the people in the house were agitated and the pregnant woman’s husband had stood up to hurt me.” (ASHA 12)

Theme 2: experiences of ASHA workers

Category 2.1: Response From Postnatal Mothers and the Community

The majority of ASHA workers had good experiences during postnatal home visits. Families had positive responses to their visits. ASHAs mentioned that they often forgot to make PHC visits, but mothers and their families reminded them to do so. ASHAs also attended deliveries with mothers and visited the newborn if the child was admitted to a hospital. ASHAs assisted postnatal mothers with documentation which is useful in obtaining financial benefits from the government:

“A pregnant lady started suffering from labor pain. Her husband was at home. We took the pregnant woman to the hospital after the husband called me for delivery. There, the doctor said that delivery would have to be done by operation. On hearing this, her husband started crying, asking, now if the girl comes again, too? We assured him not to worry; everything would be fine. The operation was done, and the boy was born. Even today, her husband respects us.’’ (ASHA 6)

Theme 3: reason for not reaching in 24 hours in case of home delivery

Category 3.1: Home Delivery Conducted by a Trained Dai

The majority of ASHAs mentioned that home deliveries used to be common earlier. Currently, however, the majority of deliveries took place at institutions such as hospitals or PHCs. Few pregnant women delivered babies at home. A traditional birth attendant conducted almost all deliveries at home. Traditional birth attendants told the ASHA about the delivery the next day. This was the reason for the delay by the ASHA in making her first visit to see the mother and the newborn:

“Right now, no home delivery here. One occurred many years ago. They were taken to the PHC. They said there was still time for delivery. Then we came home, and the delivery was done. It was the third delivery. Her mother-in-law conducted the delivery at home. Her mother-in-law is a trained dai. I found out the next morning when I went to get the water at handpump. They informed me about the delivery. Then we took the mother and newborn to the PHC.” (ASHA 1)

Theme 4: support from healthcare workers

Category 4.1: Response From Staff

Most ASHA workers received support from FHWs, the Community Health Officer, the ASHA facilitator, the medical officer, and the Block Health Officer. FHWs and medical officers assisted them in referring postnatal mothers or high-risk and low-birth-weight babies. When FHWs were occupied with their duties, ASHA workers managed on their own:

“ASHA facilitator helps us. A high-risk pregnant woman was taken to the hospital for delivery. From there, she was asked to be referred to a higher hospital. I took her for delivery at a private hospital, where they said that she would need four bottles of blood. Block Health Officer helped in this matter.” (ASHA 5)

Theme 5: community issue

Category 5.1: Type of Response

A few people occasionally refused postnatal care services. Additionally, some people were worried about healthcare workers visiting them at home during the COVID-19 pandemic:

“Some individuals [were] opposed to receiving services and asked, why are you here? We have other work; we have to go for work. There are 2-5% of such people.” (ASHA 8)

Category 5.2: Breastfeeding Issue

Some ASHAs said that if a cesarean birth occurs, they cannot advise that the mother start breastfeeding because of the side effects of anesthesia. They also mentioned that if mothers were uncomfortable speaking in front of men, they could visit courtyard shelter (Aanganwadi Centre) where ASHAs could offer advice on how to breastfeed. One ASHA mentioned a mother who had not eaten on the day of her delivery and only started breastfeeding on the second or third day after delivery. Another ASHA stated that in a private hospital, a healthy newborn is kept for a day. Nursing care begins the next day. They advised the mother to warm up some cow’s milk and feed it to the child with a spoon. If the mother was sick, she would not be able to produce milk. In such cases, some societies believed that newborns should be fed goat’s milk. Some families also had a tradition of boiling powdered milk or milk from cows or goats, removing the cream from the milk, and feeding the milk to the baby:

“It is advised to begin breastfeeding as soon as possible after delivery in a government hospital; however, if a cesarean surgery is performed in a private hospital, the mother is not permitted to do so, and they have given powdered milk. The infant shouldn’t be allowed to start breastfeeding if the mother has received anesthesia. That’s why the baby is fed powdered milk. She breastfeeds her child the next day.” (ASHA 1)

Category 5.3: Prelacteal Feeding

Most ASHAs claimed that members of the community formerly practiced prelacteal feeding. This was common when deliveries occurred at home. Today, however, the majority of deliveries take place in hospitals. Mothers in joint families practice prelacteal feeding more frequently, such as by giving the child jaggery water. ASHAs claimed that in-laws’ input was often the reason behind practicing prelacteal feeding. Prelacteal feeding was linked to inadequate information, lifestyle in a joint household, home deliveries, and cesarean sections.

Category 5.4: Myths and Taboos on Family Planning and Contraception

According to ASHAs, there are many misconceptions and false assumptions in the community regarding spacing methods. There are some widespread misconceptions regarding oral contraceptive pills (e.g., they promote weight gain) and intrauterine contraceptive devices (they may result in infections or may travel up to the chest cavity and create issues). Both men and women shared concerns about tubal ligation. Cultural beliefs that prevent women from making their own decisions have a big influence on how family planning programs are implemented. The majority of ASHAs said that the husband and wife make decisions about family planning together in some families, whereas in other families, the mother-in-law makes the decision. An ASHA worker discussed family planning methods with husbands and mother-in-laws:

“We shouldn’t create any pressure on them. All of it is their wish. We provide information about copper-T. Although several mothers reported that their husbands refused, we also bring condoms with us. Then, if we explain this to their husbands, they will accept. In some families, if women are there, they are all feeling comfortable, but if there are any men, we request that they go. Contraception is less likely in use if a boy has not yet been born, and decisions regarding family size and the use of contraception frequently rely on the sex of the first child. Both husband and wife favor sons more than daughters.”

Theme 6: solution to improve postnatal care

Category 6.1: Infrastructure and Incentives

The majority of ASHA workers mentioned the need to improve infrastructure and incentives. Some claimed that they were working harder than FHWs. They wanted to become long-term government employees. They believed they were entitled to sufficient training, assistance, recognition, and remuneration for the duties they performed. They also believed that giving them psychological support was equally important. ASHAs raised the issue of reimbursement delays for their travels. Rural areas have fewer transportation options and cover larger distances, which increases the overall cost. Some ASHAs suggested opening a local radio imaging facility so that they do not have to travel far.

**Figure 2:**
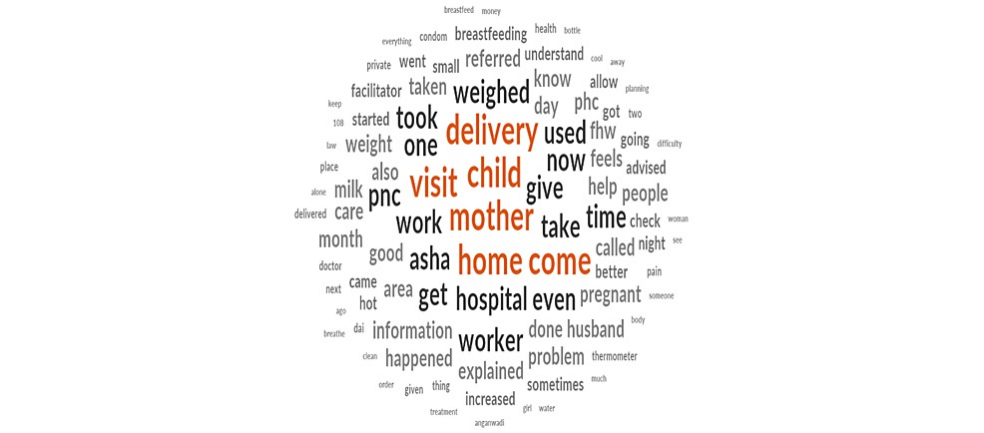
Word cloud of most frequently occurring words in the in-depth interviews of ASHAs. ASHA = Accredited Social Health Activist Image credits: Dr. Kinjal Gadhavi.

Figure 2 shows a visual representation of text data where each word’s size indicates its frequency or significance. Notable terms include “delivery,” “child,” “mother,” “visit,” “home,” “hospital,” “worker,” and “care,” indicating a predominant focus on childbirth and maternal healthcare. Furthermore, words like “visit,” “home,” and “worker” suggest home-based interventions such as postnatal home visits and postnatal services. Additionally, the prevalence of terms such as “weighed,” “milk,” “breastfeeding,” and “health” indicates that themes such as breastfeeding practices, newborn weight monitoring, and holistic health management have been included in newborn care and postnatal services. The findings provide valuable insights into prevailing concerns and areas of focus within maternal and newborn healthcare.

## Discussion

The World Health Organization advises postnatal home visits to improve the health of mothers and healthy newborns. Home visits within the first week after birth are recommended for better care of the mother and the newborn. The primary goal is to decrease maternal and infant mortality and morbidity by ensuring sustained care after hospital discharge. There is less evidence of the impact of postnatal home visits on maternal health. Included in these are newborn care, postpartum problems, behavioral changes, early childhood development, the provision of postpartum home visit kits, and the conduct of home visits between 48 to 72 postnatal hours. These visits are intended to assist in the early detection of physical and mental health issues, rapid referral, preventive and physical exercise, hygiene, responsive parental roles, family planning, nutrition and breastfeeding, and promoting follow-up visits to the health center for postnatal examination and immunization [11].

This strengthens community practices for newborn care, early detection of neonatal disease, and appropriate referral through home visits. ASHAs, who work as front-line healthcare workers and are responsible for providing preventative care services to mothers and their newborns, are expected to deliver services. Service delivery through home-based newborn care was largely limited to health education and referral. A low level of efficiency has been observed in providing neonatal care services [12].

The main goal of the study was to assess the optimal postnatal care of mothers and newborns in a rural area and identify gaps in a qualitative study. According to ASHAs, postnatal mothers believed the care was satisfactory. The areas that needed improvement were expanding the services offered and improving training in communication skills and social acceptance. The needs of the postnatal mother and her newborn should be taken into consideration.

This study revealed a lack of transportation services for ASHAs to reach the headquarters, i.e., PHCs. There was no support system to minimize the challenges of ASHAs related to transportation. A similar observation was made in rural China that poor road conditions, longer distances, and separated communities made PHC service difficult for providers to visit women at home. Low coverage and average postnatal care represent a neglected part of maternity services and a gap in the continuum of care [13]. Transportation issues listed in other studies include personal expenditures, far-off areas, transportation by sharing autorickshaws, and walking with a heavy bag [14].

In low and middle-income countries, financial remuneration is key for motivating ASHA performance. According to the current study, payment delays were quite common. The study further revealed high workloads and poor incentives were the two key issues that impacted the quality of maternity care. Most ASHAs had low socioeconomic backgrounds, and the incentives were the only source of income for their families. In addition, it was observed that these incentives were delayed when documentation was inadequate, incorrect, or submitted late. Studies have found that ASHAs find it difficult to manage work and home obligations when they work more than 3.5 hours a day [8]. A similar observation was made in a study conducted among midwives in Indonesia [15].

The study also revealed that inadequate materials and resources were directly impeding the ability of ASHAs to complete their tasks. A similar observation was noted in another study that showed that a lack of resources adversely impacts the effectiveness of community healthcare workers [16].

The study revealed that few villagers were using abusive language or insulting behavior toward ASHA workers, which ultimately resulted in non-compliance with ASHA work output and poor interpersonal communication. Gogoi et al. also reported that despite communities using such languages, ASHAs perform their jobs positively [17].

Prelacteal feeding was prevalent among uneducated mothers and home-delivered babies in the past, as reported by AHSAs in this study. Honey and jaggery water were the most common prelacteal feeds. Currently, this practice is rare. Asim et al. (2020) reported that mothers gave prelacteal feed to both male and female newborns, regardless of their age or area of residence. Additionally, there was no difference in prelacteal feeding between mothers with higher education and those who were illiterate [18].

## Conclusions

Postnatal mothers have received considerably less attention than antenatal mothers because it mostly depends on ASHA workers and field staff. ASHA workers are doing their best regarding postnatal care. This study revealed a few issues such as logistical issues, transportation issues, regular and timely payment issues, and local-level acceptance issues. If PHC medical officers and local villagers support ASHAs in dealing with these problems, it will improve the quality of care. These problems are minor and can be solved at the local level.

## Appendices

Research objective: To assess postnatal care services quality by ASHA (Accredited Social Health Activist) workers and associated factors such as newborn care.

Thank you for taking the time to speak with me today. I have several questions to ask you that I have prepared in advance. I will turn on the tape recorder now. If you have any additional questions or comments as we do the interview, please feel free to share them with me.

Interview details: name of Primary Health Center, interviewer name, date of interview, interview start time, interview end time, tape recording number.

Sociodemographic characteristics: code, age, sex, occupation, religion, marital status, education, total work experiences.

Questions for ASHA: Do you have any problems while providing PNC care services? What are your experiences? Tell one good and one bad experience. Do you have adequate equipment and capacity for PNC care? What do you do when there is a problem with the equipment or the weight machine is not working? Share your experience (% of such bad experiences). In the case of home delivery, can you reach for a visit in 24 hours? What are your experiences? (training for delivery, delivery kit). During your PNC care, if you need support, who is giving you support? Do you have any trouble getting support from your healthcare staff? What are your experiences? While you are on PNC care, have you had any experience of refusal of your service? What are your experiences in this area? What are your experiences with a breastfeeding practice in your area? (Exclusive breastfeeding - top feeding, cow milk, prob for Galthuthi twice). What Do You Provide in Family Planning? How do you explain it to the mother? In front of another family member or in private? What are your experiences? (role of husband in decision making). Share your two good and bad experiences. What is your opinion on improving PNC services coverage?

## References

[1] World Health Organization (2010). WHO Technical Consultation on Postpartum and Postnatal Care. https://apps.who.int/iris/bitstream/handle/10665/70432/WHO_MPS_10.03_eng.pdf.

[2] Selvaraj R, Ramakrishnan J, Sahu SK, Kar SS, Roy G (2021). Community-based assessment of postnatal care in Puducherry-a cross-sectional study. J Family Med Prim Care.

[3] (2022). WHO recommendations on maternal and newborn care for a positive postnatal experience. https://www.who.int/publications-detail-redirect/9789240045989.

[4] (2024). Maternal health | UNICEF India. https://www.unicef.org/india/what-we-do/maternal-health.

[5] McCauley M, McCauley H, van den Broek N (2021). Essentials of postnatal care for the mother and baby. The Continuous Textbook of Women’s Medicine Series - Obstetrics Module.

[6] Manna S, Basu S (2023). It cost us all of our savings to deliver our baby: a qualitative study to explore barriers and facilitators of maternal and child health service access and utilization in a remote rural region in India during the COVID-19 pandemic. Cureus.

[7] Anuradha Anuradha, Harleen Harleen, Singh T, Maji D (2022). A study to evaluate the knowledge of postnatal care among accredited social health activist workers in North Indian rural area. Int J Reprod Contracept Obstet Gynecol.

[8] Manjunath U, Sarala R, Rajendra D (2022). Assessment of workload of ASHAs: a multi-stakeholder perspective study for task-sharing and task-shifting. J Health Manag.

[9] (2024). Villages and towns in Sankheda Taluka of Vadodara, Gujarat - Census India. https://www.censusindia.co.in/villagestowns/sankheda-taluka-vadodara-gujarat-3904.

[10] (2024). Online map - Gujarat all village maps | MaruGujarat.net. https://marugujarat.net/online-map-gujarat-all-village-maps/.

[11] de Vries I, Abu Hamad B, van Gurp M, Alba S, Khammash U, Baatsen P (2021). Key lessons from a mixed-method evaluation of a postnatal home visit programme in the humanitarian setting of Gaza. East Mediterr Health J.

[12] Neogi SB, Sharma J, Chauhan M (2016). Care of newborn in the community and at home. J Perinatol.

[13] Chen L, Qiong W, van Velthoven MH (2014). Coverage, quality of and barriers to postnatal care in rural Hebei, China: a mixed method study. BMC Pregnancy Childbirth.

[14] Brahmbhatt M, Sheth J (2017). Focused group discussion of urban ASHA workers regarding their work related issues. Indian J Community Health.

[15] Probandari A, Arcita A, Kothijah K, Pamungkasari EP (2017). Barriers to utilization of postnatal care at village level in Klaten district, central Java Province, Indonesia. BMC Health Serv Res.

[16] Jaskiewicz W, Tulenko K (2012). Increasing community health worker productivity and effectiveness: a review of the influence of the work environment. Hum Resour Health.

[17] Gogoi MM (2020). ASHA worker as human resource and problem faced by them before and after COVID-19. PalArch’s J Archaeol Egypt.

[18] Asim M, Ahmed ZH, Hayward MD, Widen EM (2020). Prelacteal feeding practices in Pakistan: a mixed-methods study. Int Breastfeed J.

